# *Scara3* regulates bone marrow mesenchymal stem cell fate switch between osteoblasts and adipocytes by promoting Foxo1

**DOI:** 10.1111/cpr.13095

**Published:** 2021-07-12

**Authors:** Peng Chen, Biao Hu, Ling‐Qi Xie, Tie‐Jian Jiang, Zhu‐Ying Xia, Hui Peng

**Affiliations:** ^1^ Department of Endocrinology Endocrinology Research Center Xiangya Hospital of Central South University Changsha China; ^2^ Department of Orthopedic Xiangya Hospital of Central South University Changsha China

**Keywords:** adipocytes, BMSCs, osteoblasts, osteoporosis, *Scara3*

## Abstract

**Objectives:**

Scavenger receptor class A, member 3 (*Scara3*) was involved in adipogenesis. However, the effect of *Scara3* on the switch between osteogenesis and adipogenesis of bone marrow mesenchymal stem cells (BMSCs) remains elusive.

**Materials and Methods:**

The correlations between *SCARA3* with the osteogenic‐related were analysed based on the GTEx database. The effects of *Scara3* on osteogenic or adipogenic differentiation of BMSCs were evaluated by qPCR, Western blot (WB) and cell staining. The mechanisms of *Scara3* regulating Foxo1 and autophagy were validated by co‐expression analysis, WB and immunofluorescence. In vivo, *Scara3* adeno‐associated virus was injected into intra‐bone marrow of the aged mice and ovariectomized (OVX) mice whose phenotypes were confirmed by micro‐CT, calcein double labelling and immunochemistry (HE and OCN staining).

**Results:**

*SCARA3* was positively correlated with osteogenic‐related genes. *Scara3* expression gradually decreased during adipogenesis but increased during osteogenesis. Moreover, the deletion of *Scara3* favoured adipogenesis over osteogenesis, whereas overexpression of *Scara3* significantly enhanced the osteogenesis at the expense of adipogenesis. Mechanistically, *Scara3* controlled the cell fate by promoting Foxo1 expression and autophagy flux. In vivo, *Scara3* promoted bone formation and reduced bone marrow fat accumulation in OVX mice. In the aged mice, *Scara3* overexpression alleviated bone loss as well.

**Conclusions:**

This study suggested that *Scara3* regulated the switch between adipocyte and osteoblast differentiation, which represented a potential therapeutic target for bone loss and osteoporosis.

## Graphical abstract

Aberrant autophagy caused the pathogenesis of osteoporosis, partially associated with the increase of osteogenic differentiation and decrease of adipogenic differentiation in BMSCs. However, *Scara3* can promote the Foxo1 expression and autophagy flux, thus alleviate the bone loss and bone marrow adipose tissue accumulation.

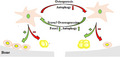

## 1 INTRODUCTION

Osteoporosis is characterized by low bone mass and microarchitectural dysfunction.^1^ Bone marrow mesenchymal stem cells (BMSCs) have potentials to differentiate into adipocytes and osteoblasts, whose cell fate was altered in osteoporotic individual.^2^, ^3^, ^4^, ^5^ Accumulated studies identified various master regulators associated with the adipogenic and osteogenic lineage commitment.^6^, ^7^ Some non‐coding RNA, such as *miR‐188* and *Bmncr*, were declining with ageing, which both contributed to reduced bone mass and increased marrow adipose tissue (MAT) through controlling the BMSCs fate.^8^, ^9^ Other studies showed various protein modulators were involved in the cell fate switch of BMSCs, such as RB, PGC‐1α and FOXP1.^10^, ^11^, ^12^ However, the molecular network elucidating the bone‐fat balance still needs further investigations.

*SCARA3* encodes a macrophage scavenger receptor‐like protein which is ubiquitously expressed in human tissues.^13^ *SCARA3* was reported to protect cells from oxidative stress‐induced cell damage through removing oxidizing molecules or harmful products of oxidation.^13^, ^14^ Han et al^13^ showed that the SCARA3 expression could be promoted upon the oxidative stress stimulation. However, the increase of SCARA3 expression diminished when there was excessive oxidative stress. Several disorders, such as obesity and multiple myeloma, characterized by a high level of oxidative stress, displayed a lower expression of SCARA3.^15^, ^16^ In addition, *SCARA3* was recognized as a tumour suppressor‐related gene because of its downregulation in prostate cancer tissues and involvement in cancers metastases and progression.^14^, ^15^ Through analysing a data set obtained from GEO database (GSE115068),^17^ we previously found that the expression of *Scara3* was downregulated in Ad‐MSCs isolated from young mice than from old mice.^16^ Moreover, our previous study showed that the *SCARA3* gene was a critical regulator of adipogenic differentiation.^16^ Balla et.al^18^, ^19^ demonstrated that *SCARA3* transcription was significantly downregulated in osteoporosis bone tissue, but they did not further explored the functional roles of SCARA3 in bone formation. Based on these evidence, we investigated whether *Scara3* played a role in controlling the bone‐fat balance.

Autophagy, as a major catabolic process responsible for the degradation of damaged macromolecules and organelles, is now well‐established as a regulator of bone physiology and bone‐related disorders.^20^, ^21^, ^22^, ^23^, ^24^ Previous studies showed that autophagy can affect the differentiation of osteoblasts and osteoclasts.^21^ Moreover, aberrant autophagy could result in the dysfunction of cell fate in BMSCs.^20^, ^25^ Liu et.al^26^ recently showed that autophagy receptor OPTN (optineurin) regulated bone‐fat balance by regulating the cell fate switch. However, it is unclear that if *SCARA3* gene is involved in autophagy.

In this study, we revealed *Scara3* as an important modulator that favoured osteogenic differentiation over adipogenic differentiation. *Scara3* plays an important role in autophagy signalling through regulating Foxo1. Moreover, we found that overexpression of *Scara3* through intra‐bone marrow injection of AAVs‐*Scara3* can lead to the increase of bone formation and decrease of bone marrow fat in ovariectomy (OVX) mice. Together, our study reported a new potential target to prevent and treat osteoporosis.

## 2 METHODS AND MATERIAL

### 2.1 Bioinformatic analysis

Genotype‐Tissue Expression (GTEx) program (https://www.gtexportal.org/) is a research project of normal tissue specific gene expression and regulation of comprehensive public database. We downloaded the raw genes expression data, ID conversion files, clinical phenotypes and GTF annotation file from the GTEx program for the co‐expression calculation. Next, we integrated the gene expression and GTF annotation file with clinical phenotypes and then removed the data without relevant clinical information. Based on the fact that *RUNX2*, *SP7* and *BALAP* are important regulatory genes in the process of osteogenic differentiation, we further analysed the correlation between *SCARA3* gene and three key genes of osteogenic differentiation. Pearson's correlation (*R*) and *P* values were calculated. The *P*‐value has been marked as ‘0’ when it is less than 1 ∗ 10^−8^.

BioGPS (http://biogps.org/#goto=welcome) is a free, extensible and customizable gene annotation portal that has resource for learning then gene and protein function. We extracted the expression data of *Scara3* in osteoblasts at the day 5, day 14 and day 21 from Geneatlas MOE430, GCRMA.

### 2.2 Mice

All mice with a C57/B6 background were purchased from Shanghai Slac Laboratory Animal Co. The 2‐month‐old female mice were performed the ovariectomy (OVX) surgery. Then, random allocation was taken to divide the mice after OVX. One week later, 10^11^vg AAV (adeno‐associated virus) was injected into femur bone marrow of mice in the vehicle group. For the treatment group, 10^11^vg AAV was injected into femur bone marrow. 15 month‐old male mice were also treated with control AAVs or AAVs‐*Scara3* at the same dosage using intra‐bone marrow injection. Five mice were used for each group of each independent experiment. All protocols associated mice's care and experiments used in this study were reviewed and approved by the Animal Care and Use Committees of the Laboratory Animal Research Center at Xiangya Medical School of Central South University. All animals were maintained in a specific pathogen‐free facility of the Laboratory Animal Research Center at Central South University, with free access to food and water prior to the initiation of experiments.

### 2.3 Intra‐bone marrow injection

Intra‐bone marrow injection was carried out to deliver the AAV (10^11^vg) into mice. Briefly, mice were anaesthetized. Then, the hair near the knee joints were shaved. The knee joint was exposed using the microscissors to separate muscle tissue and using the tweezers to push the tendon to left side. Then, a 29‐gauge insulin syringe was inserted into bone marrow cavity. 10^11^vg in 10‐15 μL of AAVs was delivered into the bone marrow cavity. The muscle and skin were stitched.

### 2.4 Ovariectomy

Ovariectomy surgery was performed as described before.^27^ Five mice in each group. Briefly, mice were anaesthetized. Then, the hair on the waist were shaved. The kidney and the white adipose tissue on the kidney were seen after cut the skin and peritoneum. After removal of the ovaries, the oviducts were ligated and the peritoneum and skin were sealed.

### 2.5 µCT analysis

Femora were dissected from mice, and the muscle and other tissue were carefully removed. The bone tissues were then fixed in the 4% paraformaldehyde overnight and followed by the analysis by a high resolution Uct (Skyscan 1172; Bruker microCT). The voltage, current and resolution of scanner were set as 65 kv, 153 μA and 15 µm per pixel, respectively. The image reconstruction software (NRecon, version 1.6; Bioz), data analysis software (CT Analyser, version 1.9; Bruker microCT) and three‐dimensional model visualization software (μCT Volume, version 2.0; Bruker microCT) were applied to analyse the parameters of the distal femoral metaphyseal trabecular bone. The region of interest (ROI) was defined at the area of 5% proximal to the growth plate in the distal femora. A series results including trabecular bone volume per tissue volume, trabecular number, trabecular separation and trabecular thickness were obtained.

### 2.6 Calcein double labelling

The mice were treated with calcein at the dose of 0.5 mg/per mice (Sigma‐Aldrich) using intraperitoneal injection. The first injection was carried out at 10th day before euthanasia, and the second injection was given at the second day before euthanasia. The femora were dissected and fixed in the 4% paraformaldehyde overnight. Calcein double labelling was performed in the 5 μm longitudinal sections of undecalcified bone slice to evaluate MAR and BFR using Image‐Pro Plus 6.0. Four randomly chosen visual fields in the distal metaphysis of the femur were measured to test trabecular bone formation in femora. The images were captured by a fluorescence microscope (Leica).

### 2.7 Histochemistry and histomorphometry analyses

Femora were dissected from mice and fixed with 4% PFA (Paraformaldehyde) overnights, followed by the decalcification in 14% EDTA for 1 week. Then, decalcified bones were embedded in the paraffin and cut into 4‐μm‐thick sections. To assess the capacity of bone formation, the OCN (Abcam) staining and HE staining were performed on the bone tissue slice according to the previous described method.^27^ Briefly, for the OCN staining, bone sections were processed for antigen retrieval followed by the blocking by 3% BSA with Triton 100. Then, the bone sections were incubated by the primary antibody against osteocalcin (catalog M173; Takara) overnight at 4°C. Next, HRP‐DAB cell from a tissue staining kit was used to detect the immunoactivity according to the manufacturer's instruction. For the HE staining, bone sections were conducted according to standard protocol. Briefly, sections were stained by haematoxylin for 30 seconds and eosin for 3 minutes.

### 2.8 Bone marrow mesenchymal stem cells isolation and culture

Mouse BMSCs cell line (MUBMX‐01001; Cyagen Biosciences) was cultured in Mouse Mesenchymal Stem Cell Growth Medium (MUBMX‐90011; Cyagen Biosciences). Primary Mouse BMSCs were isolated from 1‐month‐old male C57/B6 mice according to the previous described method.^8^, ^9^ Briefly, mice were anaesthetized using 5 μL/g 5% pentobarbital sodium. Then, the femurs and tibias were dissected from mice and put in the PBS with 100 U/mL penicillin and 100 µg/mL streptomycin. After washing the bone tissues in the PBS with 100 U/mL penicillin and 100 µg/mL streptomycin three times, the both ends of bones were cut off and the bone marrow were flushed out through culture medium in syringe. Then, the cells were cultured in DMEM with 5% FCS supplement with 100 U/mL penicillin and 100 µg/mL streptomycin overnight. Next day, the non‐adherent cells were removed using PBS to obtain purified BMSCs.

### 2.9 Cell transfection

The *Scara3* siRNA/*Foxo1* siRNA and the negative control (NC) were purchased from Ribibio. The m*Scara3* pcDNA3.1‐3xFlag‐C construct, LC3‐GFP construct and negative control were purchased from YouBao Biology. 100 nmol/L siRNAs or 1ug plasmid were transfected into BMSCs using lipofectamine 2000 (Invitrogen) according to manufacturer's recommendations.

### 2.10 Osteogenic differentiation and mineralization assay

To induce osteogenic differentiation of BMSCs, primary BMSCs with or without previous treatments were cultured in 6‐well plates at 2.5 × 10^6^ cells per well with the mesenchymal stem cell osteogenic‐induced medium (10% FBS, 100 U/mL penicillin, 100 µg/mL streptomycin, 0.1 mmol/L dexamethasone, 10 mmol/L β‐glycerol phosphate and 50 mmol/L ascorbate‐2‐phosphate). The culture medium was changed every 3 days.

After 2 days of osteogenic differentiation, the cell lysates were washed using PBS followed by homogenized for ALP activity assay by spectrophotometric measurement of p‐nitrophenol release using an Alkaline Phosphatase Assay Kit according to the manufacturer's instructions (P0321S, Beyotime).

After 7 days of osteogenic differentiation, alkaline phosphatase staining (ALP staining) was carried out to assess the capacity of mineralization as described before.^8^ Briefly, the washed cells were fixed in 10% paraformaldehyde for 5 minutes. Then, cells were incubated in ALP incubation buffer (0.2 g barbital sodium, 0.4 g magnesium sulphate, 0.2 g calcium chloride and 0.3 g beta‐glycerophosphate, 10 mmol/L β‐glycerol phosphate and 50 mmol/L ascorbate‐2‐phosphate) at 37°C for 2 hours. Next, 2% calcium chloride was used to wash the cells and 2% cobaltous nitrate was used to incubate cells for 5 minutes. Then, cells were incubated in 1:80 ammonium sulphate for 10 seconds.

After 21 days of osteogenic differentiation, Alizarin Red staining was performed according to the manufacturer's instructions (MUBMX‐90021; Cyagen Biosciences). Briefly, cells were washed using PBS three times followed by 4% paraformaldehyde for 30 minutes. After washed by PBS for three times, cells were stained in Alizarin red solution at 37°C for 5 minutes. The images were captured by the microscope.

### 2.11 Adipogenic differentiation and oil red staining

To induce adipogenic differentiation of BMSCs, primary BMSCs with or without previous treatments were cultured in 6‐well plates at 2.5 × 10^6^ cells per well with the mesenchymal stem cell adipogenic‐induced medium (MUBMX‐90031; Cyagen Biosciences). The culture medium A and B were alternately used every 3 days.

Oil red staining was performed at the 12 days of adipogenic differentiation. Briefly, cells were washed using PBS three times followed by 4% paraformaldehyde for 5 minutes. After washed by PBS for three times, cells were stained in oil red solution at 37°C for 3 minutes (3 mL Oil red was dissolved in 2 mL PBS). The stained cells were observed using the microscope.

### 2.12 qRT‐PCR analysis

For analysis of mRNA expression, RNA of cultured cells from six‐well‐plate was extracted using 1ml TRIzol reagent. 1000 ng of RNA was reverse‐transcribed into first‐strand cDNA using the Reverse Transcription Kit (Accurate Biology). Primers were designed in UCSC database or acquired from ORIGEN database. All primers have been blasted and tested their efficiency. SYBR Green PCR Master Mix (Takara) was used to perform qPCR. mRNA expression was normalized to the reference gene *Gapdh*.

### 2.13 Western blot

The protein was lysed using the mixture of RIPA, protease inhibitor (1:100). Western blot was conducted as described before.^28^ The nuclear protein was extracted according to the instruction of manufacturer. The primary antibodies, SCARA3 mAb (Sigma, SAB2106762), FOXO‐1A/LC3/P62/PPARG/FABP4 Ab(CST, #14952,#2775,#5114,#2120), RUNX2/SP7 Ab (Abcam, ab76956, ab22552), GAPDH Ab (ORIGENE, TA802519), were incubated overnight at 4℃, followed by the incubation with appropriate secondary antibodies for 1 hour at room temperature. The blots were visualized using ECL detection reagents.

### 2.14 Immunofluorescence

For immunofluorescence analysis, after the different treatment, the BMSCs cell line was washed by phosphate‐buffered saline (PBS) three times. Then, cells were fixed in 4% paraformaldehyde for 15 minutes. After the permeabilization and blocking through incubation with 5% FCS, 1% TritonX100 in PBS for 30 minutes at room temperature, the primary antibodies of FoxO1 Mouse mAb (#14952, CST,1:200) were incubated in 5% BSA in PBS overnight at 4°C and followed by Alexa Fluor R 488 Goat Anti–rabbit (ThermoFisher Scientific) at a dilution of 1:400. The slides were mounted by antifade with DAPI. Images were visualized on Olympus microscope using cellSense Dimension software or confocal microscope. To observe the LC3 punches, the cells overexpressing LC3‐GFP protein were fixed in 4% paraformaldehyde for 15 minutes followed by mount of antifade with DAPI. The images were captured by a confocal laser scanning microscope (Leica).

### 2.15 Statistical analysis

Data were imported into Excel and scaled and normalized to appropriate controls. Unpaired, two‐tailed Student's *t* test was performed for the comparisons of two groups. One‐way ANOVA was performed for the comparison for multiple groups.

### 2.16 Ethics statement

The animal study was reviewed and approved by Xiangya Hospital of Central South University of ethics committee.

## 3 RESULTS

### 3.1 *Scara3* is a critical modulator involved in adipogenesis and osteogenesis of BMSCs

**FIGURE 1:**
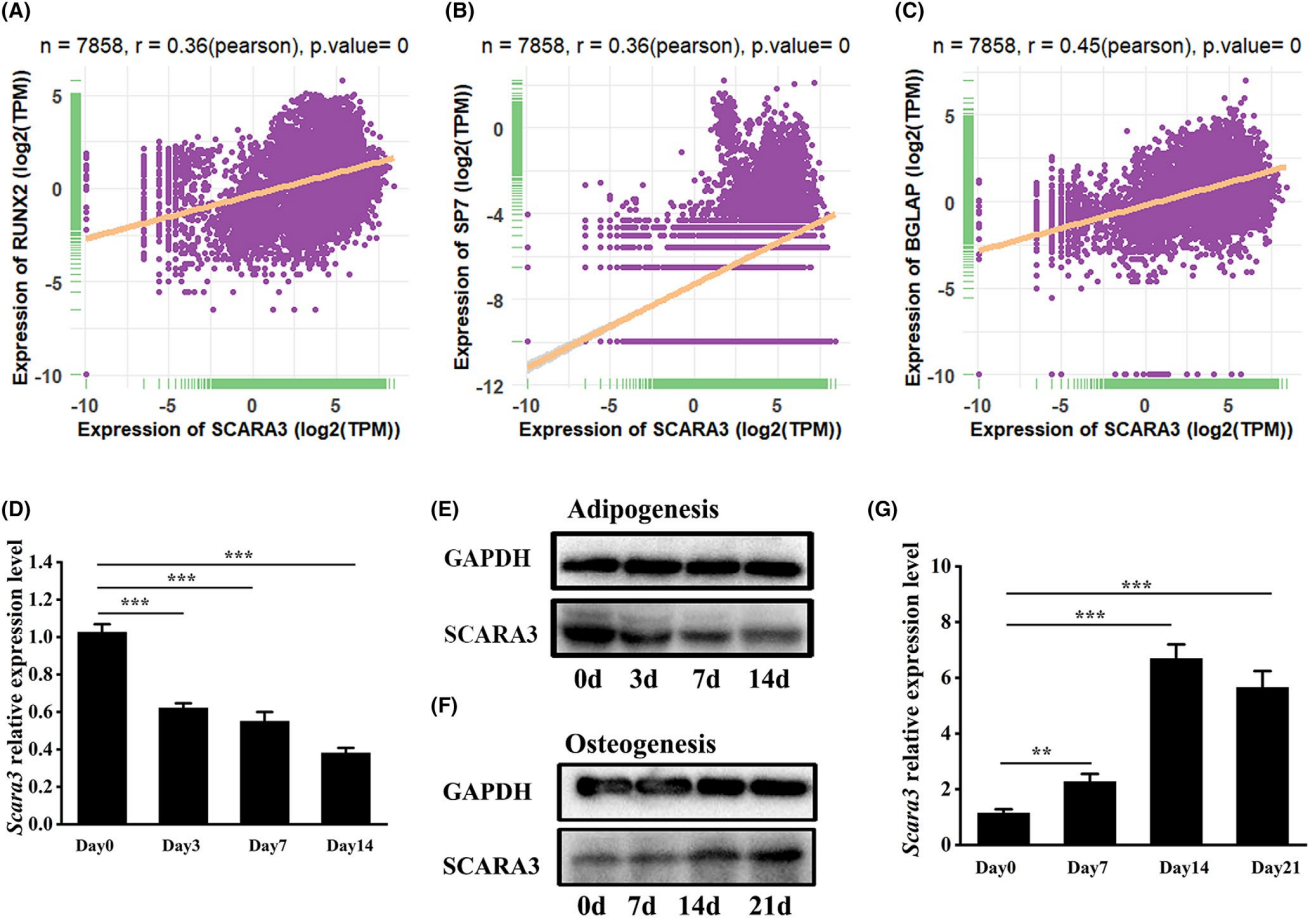
*SCARA3* was associated with the balance between adipogenic differentiation and osteogenic differentiation. (A–E) Correlation of *SCARA3* with *RUNX2* (A), *SP7* (B) and *BGLAP* (C) expression in 7858 types of tissues, based on data from the Genotype Tissue Expression (GTEx) database, respectively. *r* represents Pearson's correlation. (D‐E) The mRNA expression (D) and protein expression (E) of *Scara3* in BMSCs during adipogenic differentiation from day 0 to day 14. (F‐G) The mRNA expression (F) and protein expression (G) of *Scara3* in BMSCs during osteogenic differentiation from day 0 to day 21. Each group n = 3. All data are expressed as mean ± SD. **P < .01, ***P < .001, ANOVA

Bone marrow mesenchymal stem cells have multiple potential capacities to differentiate into osteoblasts and adipocytes, which is involved in the fat‐bone balance.^8^, ^29^, ^30^ To identify the key modulators of adipogenic and osteogenic differentiation, we firstly analysed the correlation between the differentially expressed genes with osteogenic‐related and adipogenic‐related genes. Based on our previous analysis suggesting that *SCARA3* was highly negatively correlated with the expression of adipogenic‐related genes,^16^ we then investigated its correlation with osteogenic‐related genes. The bioinformatics analysis revealed that *SCARA3* is positively correlated with osteogenic‐related genes, such as *RUNX2* (Pearson's *r* = .36, *P* = 0), *SP7* (Pearson's *r* = .36, *P* = 0) and *BGLAP* (Pearson's *r* = .45, *P* = 0) in 7858 types of tissues (Figure 1A‐C). As expected, we found that the expression of *Scara3* was remarkably downregulated in BMSCs during adipogenesis (Figure 1D,E). RT‐PCR and WB results also verified that *Scara3* expression was upregulated during osteogenesis (Figure 1F,G, Figure [Link], [Link]).

### 3.2 The deficiency of *Scara3* inhibited osteogenic differentiation and enhanced adipogenic differentiation

**FIGURE 2:**
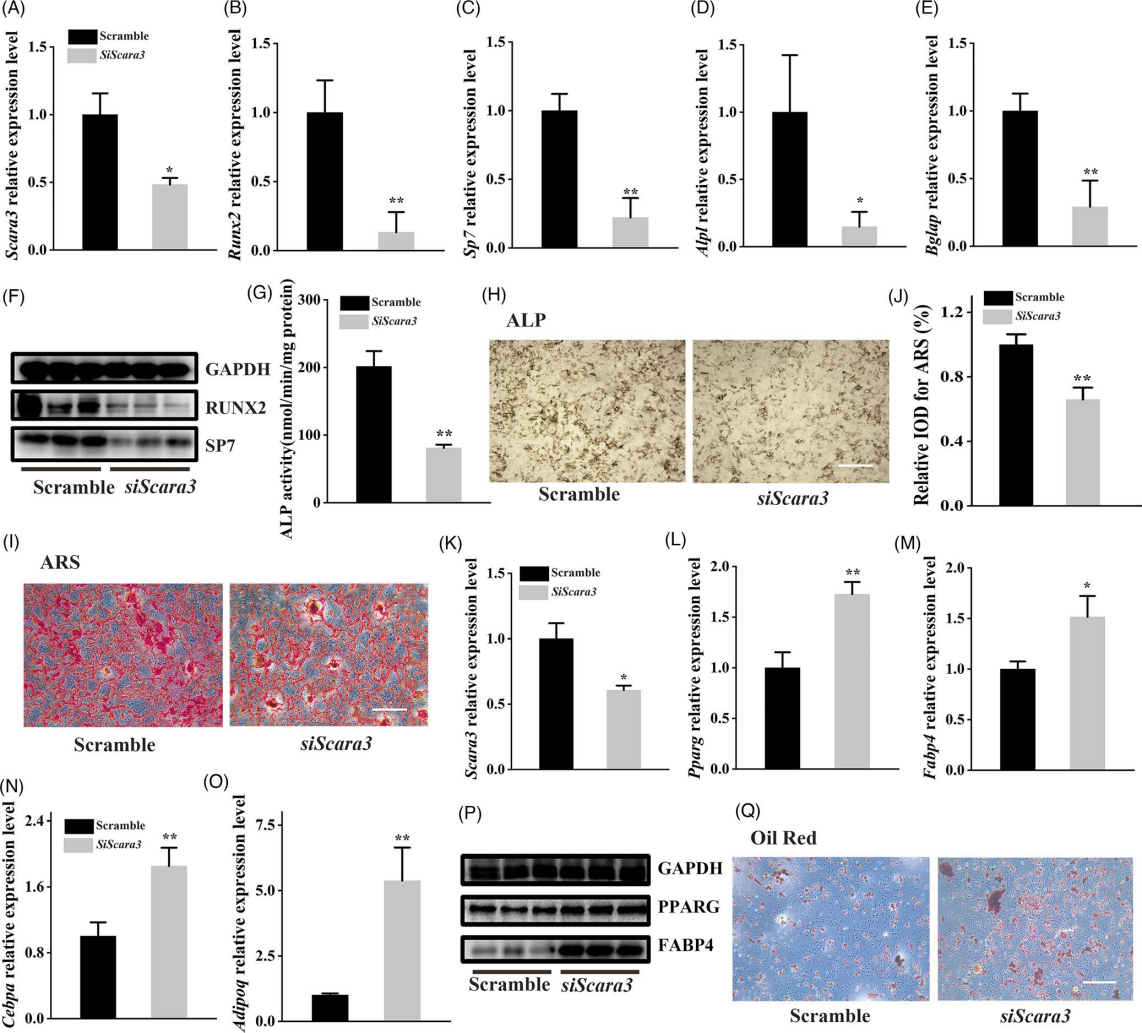
Depletion of *Scara3* impaired osteogenesis while promoted adipogenesis. (A‐J) BMSCs interfered with control siRNA or *Scara3* siRNA were followed by the osteogenic induction. (A) The mRNA expression level of *Scara3* in BMSCs. (B‐E) The mRNA expression level of osteogenic‐related genes, such as *Runx2* (B), *Sp7* (C), *Alpl* (D) and *Bglap* (E). (F) The protein expression levels of key osteogenic‐related transcriptions, including RUNX2 and SP7. GAPDH was used as a loading control. (G) Alkaline phosphatase (ALP) activity. (H) Representative image of ALP staining. Scale bar = 500 µm. (I) Representative image of Alizarin Red S (ARS) staining. Scale bar = 500µm. (J) Semi‐quantitative staining of ARS. (K‐Q) BMSCs interfered with control siRNA or *Scara3* siRNA were followed by the adipogenic induction. (K) The mRNA expression level of *Scara3* in BMSCs. (L‐O) mRNA expression levels of adipogenesis‐related genes *Pparg* (L), *Fabp4* (M), *Cebpa* (N) and *AdipoQ* (O). (P) The protein expression levels of key adipogenic‐related transcriptions, including PPARγ and FABP4. GAPDH was used as a loading control. (Q) Representative image of oil red staining. Scale bar = 500 µm. Each group n = 3. All data are expressed as mean ± SD. *P < .05, **P < .01, Student's *t* test

To investigate the role of *Scara3* in the cell fate switch of BMSCs, we silenced the expression of *Scara3* in the primary BMSCs using the *Scara3* siRNA. The knockdown efficiency measured by RT‐PCR showed the *Scara3* siRNA has been successfully transfected in BMSCs during osteogenesis (Figure 2A). Then, BMSCs with a deficiency of *Scara3* were cultured in osteogenic differentiation medium or adipogenic differentiation medium. The RT‐PCR results showed that the osteogenic markers, such as *Bglap*, *Alpl*, *Sp7* and *Runx2*, were significantly downregulated at both the RNA and protein levels in response to osteogenic induction (Figure 2B‐F). The ALP activity also suggested that the osteogenic differentiation ability was impaired by the knockdown of the *Scara3* gene (Figure 2G). The reduced mineralization was consistently validated by the ALP staining and Alizarin red staining (Figure 2H‐J). However, in response to adipogenic induction, BMSCs with *Scara3* deficiency showed higher expression of the adipogenic markers, including *Fabp4*, *Pparg*, *Cebpa* and *AdipoQ*. (Figure 2K‐P). Consistently, oil red staining showed adipogenic differentiation capacity has elevated in BMSCs after *Scara3* depletion (Figure 2Q).

### 3.3 The overexpression of *Scara3* promoted osteogenic differentiation and suppressed adipogenic differentiation

**FIGURE 3:**
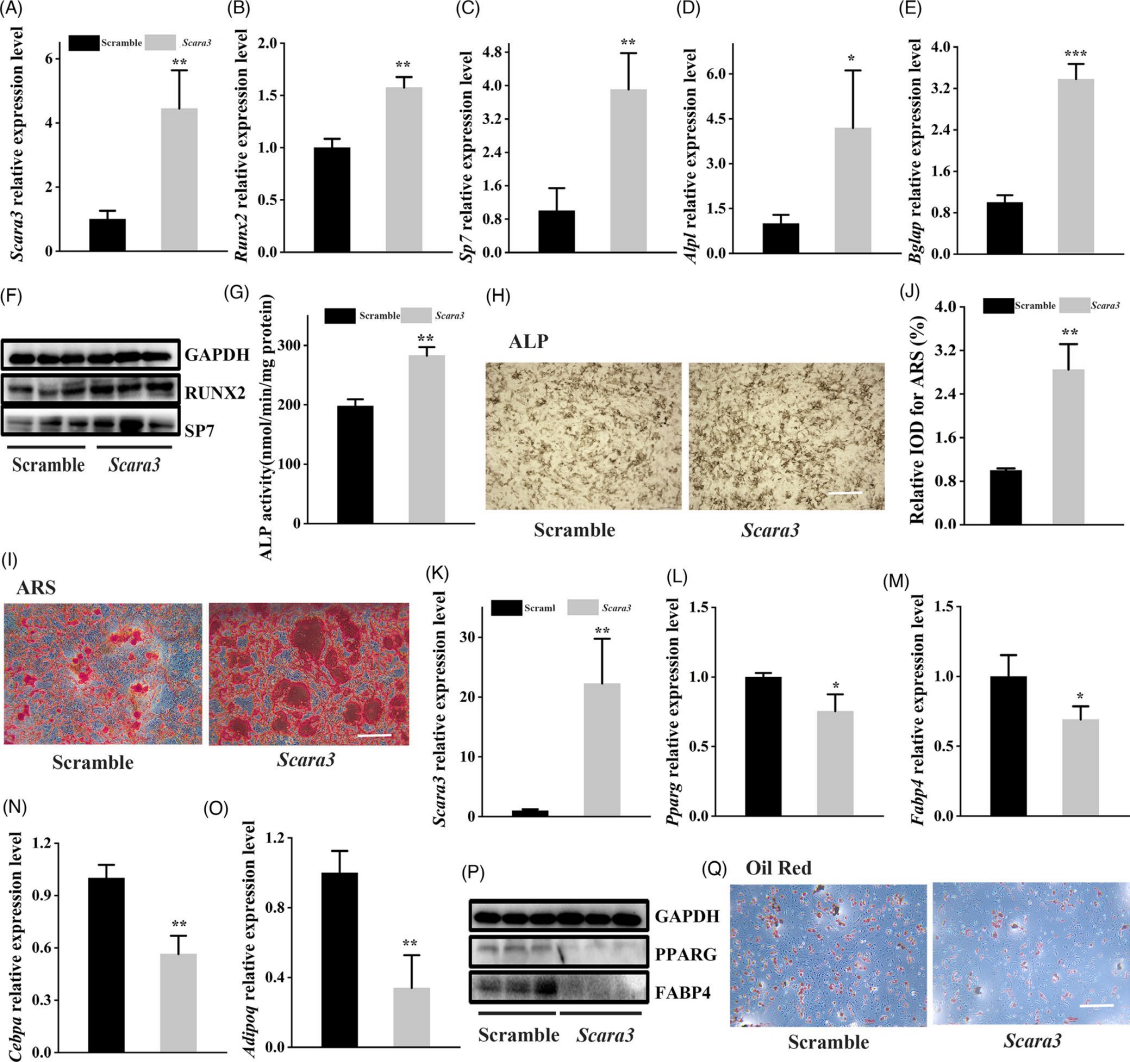
Overexpression of *Scara3* enhanced osteogenesis at the expense of adipogenesis. (A‐J) BMSCs interfered with control plasmid or *Scara3* plasmid were followed by the osteogenic induction. (A) The mRNA expression level of *Scara3* in BMSCs. (B‐E) The mRNA expression level of osteogenic‐related genes, such as *Runx2* (B), *Sp7* (C), *Alpl* (D) and *Bglap* (E). (F) The protein expression levels of key osteogenic‐related transcriptions, including RUNX2 and SP7. GAPDH was used as a loading control. (G) ALP activity. (H) Representative image of ALP staining. Scale bar = 500µm. (I) Representative image of ARS. Scale bar = 500 µm. (J) Semi‐quantitative staining of ARS. (K‐Q) BMSCs interfered with control siRNA or *Scara3* siRNA were followed by the adipogenic induction. (K) The mRNA expression level of *Scara3* in BMSCs. (L‐O) mRNA expression levels of adipogenesis‐related genes *Pparg* (L), *Fabp4* (M), *Cebpa* (N) and *AdipoQ* (O). (P) The protein expression levels of key adipogenic‐related transcriptions, including PPARγ and FABP4. GAPDH was used as a loading control. (Q) Representative image of oil red staining. Scale bar = 500 µm. Each group n = 3. All data are expressed as mean ± SD. *P < .05, **P < .01, ***P < .001, Student's *t* test

To further validate the role of *Scara3* gene in the cell lineage commitment of BMSCs, we also overexpressed *Scara3* gene using *Scara3* plasmid. The *Scara3* plasmid has been efficiently transfected in the primary BMSCs during osteogenic differentiation (Figure 3A). Then, we induced these cells to differentiate into osteoblasts or adipocytes using osteogenic differentiation medium or adipogenic differentiation medium. The results orchestrated that the osteogenic markers, such as *Bglap*, *Alpl*, *Sp7* and *Runx2*, had higher expression of BMSCs transfected with *Scara3* plasmid than control BMSCs (Figure 3B‐F). The enhancement of osteogenic differentiation ability was also supported by ALP activity (Figure 3G), ALP staining (Figure 3H) and Alizarin red staining (Figure 3I,J). However, after the induction of adipogenic differentiation, BMSCs overexpressing *Scara3* showed a remarkable decrease of adipogenic markers, such as *Fabp4*, *Pparg*, *Cebpa* and *AdipoQ*. (Figure 3K‐P). Consistently, oil red staining favoured that lipid droplet formation was inhibited by overexpression of *Scara3* in BMSCs (Figure 3Q).

### 3.4 *Scara3* regulated autophagy and controlled cell fates via Foxo1

**FIGURE 4:**
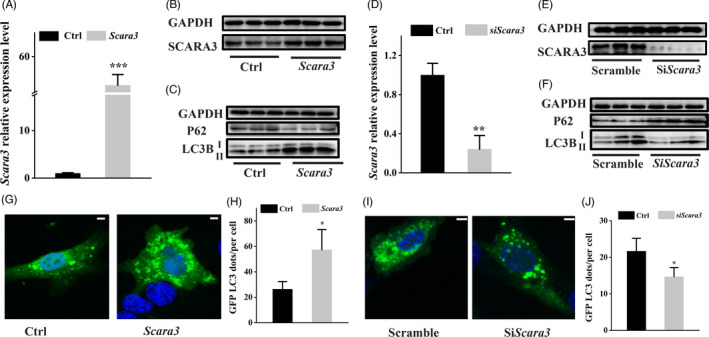
*Scara3* was involved in the autophagy process. (A‐C) BMSCs were interfered with control plasmid or *Scara3* plasmid. (A) The mRNA expression level of *Scara3* in BMSCs. (B) The protein expression level of *Scara3* in BMSCs. (C) The protein expression levels of P62 and LC3B. GAPDH was used as a loading control. (D‐F) BMSCs were interfered with control siRNA or *Scara3* siRNA. (D) The mRNA expression level of *Scara3* in BMSCs. (E) The protein expression level of *Scara3* in BMSCs. (F) The protein expression levels P62 and LC3B. GAPDH was used as a loading control. (G‐H) BMSCs expressing GFP‐LC3 were transfected with control plasmid or *Scara3* plasmid. Representative confocal image of BMSCs and quantitative analysis of Lc3 dots. Scale bar = 100 µm. (I‐J) BMSCs expressing GFP‐LC3 were transfected with control siRNA or *Scara3* siRNA. Representative confocal image of BMSCs and quantitative analysis of Lc3 dots. Scale bar = 100 µm. All data are expressed as mean ± SD. *P < .05, **P < .01, ***P < .001, Student's *t* test

Autophagy plays a crucial role in bone hemostasis.^20^, ^22^ To investigate whether *Scara3* can regulate autophagy signalling, we firstly ensured that *Scara3* was overexpressed in the primary BMSCs both at mRNA and protein levels (Figure 4A,B). Simultaneously, we tested the expression level of P62 and LC3B (Figure 4C). Western blot showed that the overexpression of *Scara3* lead to the inhibition of P62, while stimulated the expression of LC3B (Figure 4C). Conversely, knockdown of *Scara3* in BMSCs inhibited autophagy signalling (Figure 4D‐F). Next, we also detected the autophagy level through transfecting the GFP‐LC3 protein in BMSCs. The immunofluorescence confocal images showed more number of LC3 punches in BMSCs transfected with *Scara3* plasmid compared to the control BMSCs (Figure 4G,H). On contrary, a less number of LC3 punches in BMSCs interfered with *Scara3* siRNA were observed than in control BMSCs (Figure 4I,J).

**FIGURE 5:**
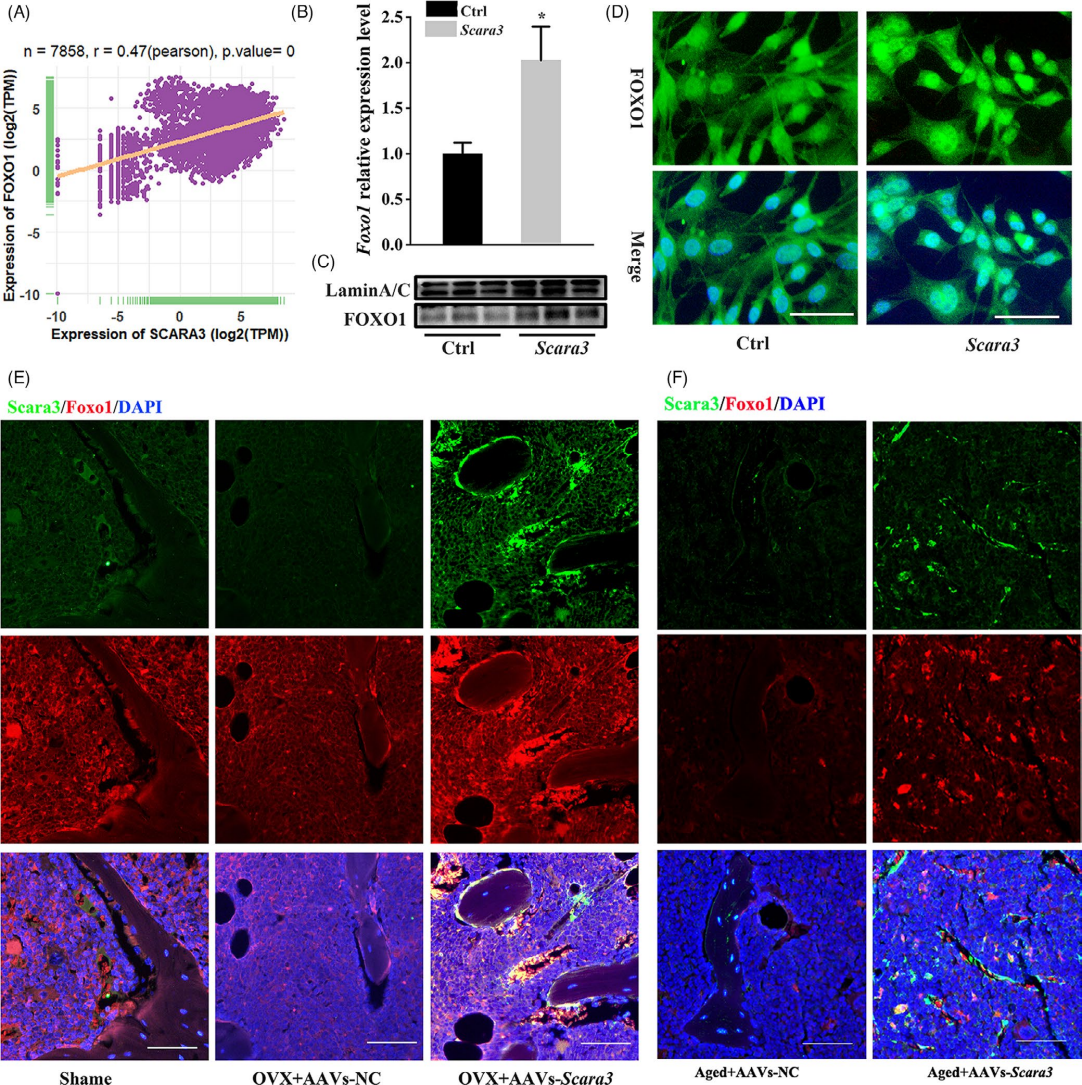
*Scara3* regulated Foxo1 expression. (A) Correlation of *SCARA3* with *FOXO1* expression in 7858 types of tissues, based on data from the GTEx database. *r* represents Pearson's correlation. (B) Foxo1a mRNA expression level in BMSCs treated with Scramble or *Scara3* plasmid. (C) FOXO1 nuclear protein expression level in BMSCs overexpressing *Scara3*. LaminA/C was used as the loading control. (D) Foxo1 immunofluorescence staining of BMSCs treated with Scramble or *Scara3* plasmid. (E) The Foxo1 expression in the femur from the aged mice overexpressing *Scara3*. DAPI staining (blue), Scara3 (green) and Foxo1 (red) were used. Scale bar = 125 µm. (F) The Foxo1 expression in the femur from the OVX mice overexpressing *Scara3*. DAPI staining (blue), Scara3 (green) and Foxo1 (red) were used. Scale bar = 125 µm

It is reported that FOXO1 can stimulate bone formation and restrain adipogenesis.^31^, ^32^, ^33^ FOXO1, as an autophagy inducer, can promote autophagy signalling as well.^34^ Then, we explored that if *Scara3* was associated with Foxos‐mediated effects. We analysed a positive correlation between *SCARA3* and *FOXO1* in 7858 types of tissues based on GTEx database. Surprisingly, the Pearson correlation was up to 0.47 (Figure 5A). In accordance with the positive correlation, the overexpression of *Scara3* in BMSCs enhanced the expression of FOXO1 (Figure 5B,C). When compared to control BMSCs, immunofluorescence images displayed that FOXO1 tended to nuclear accumulation in BMSCs with overexpression of *Scara3* (Figure 5D). In consistent with these vitro results, we found that FOXO1 expression was elevated in the 15‐month aged mice and OVX mice which were injected with AAVs‐*scara3* into intra‐bone marrow (Figure 5E,F).

**FIGURE 6:**
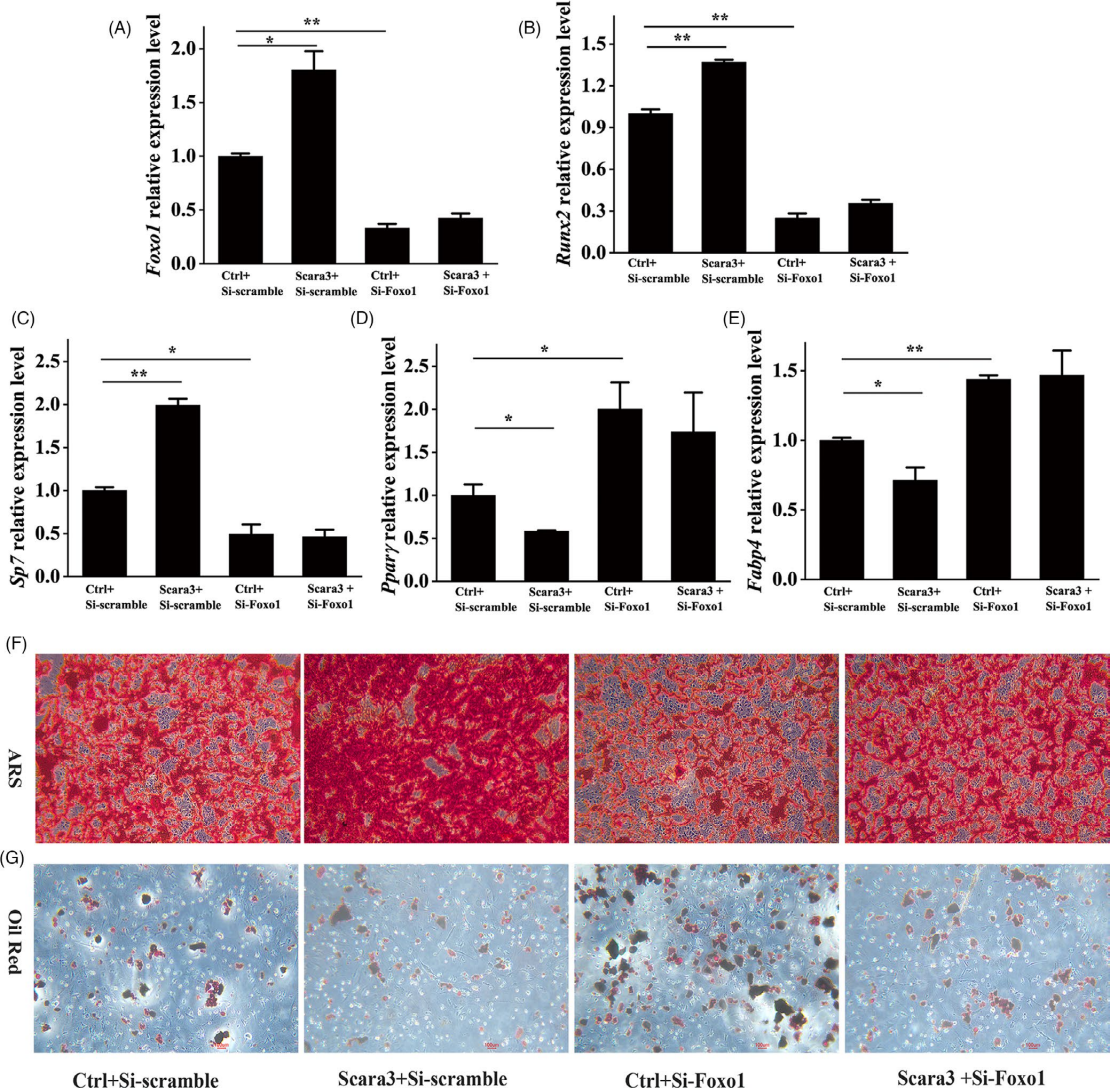
*Scara3* determined the cell fate through Foxo1. BMSCs were divided into four groups, including si‐Scaramble and Ctrl, si‐Scaramble and *Scara3* plasmids, si‐*Foxo1* and Ctrl, si‐*Foxo1* and *Scara3* plasmids, respectively. (A) Foxo1 gene mRNA expression levels in four groups of BMSCs with different treatments. (B‐C) The mRNA expression levels of osteogenic genes (*Runx2* and *Sp7*) in the 4 groups of BMSCs. (D‐E) The mRNA expression levels of adipogenic differentiation‐related genes (*Pparg* and *Fabp4*) in the four groups of BMSCs. (F) Representative image of ARS. Scale bar = 500 µm. (G) Representative image of Oil Red S staining. Scale bar = 500 µm. All data are expressed as mean ± SD. *P < .05, **P < .01, ANOVA

Next, we would like to explore whether *Scara3* determined the cell fate of BMSCs through FOXO1. Thus, BMSCs were split into four groups which were interfered with or without *Scara3* plasmids and (or) si‐*Foxo1*, respectively (Figure 6A). In comparison with the control group, upregulation of *Runx2* and *Sp7* in response to the osteogenic differentiation and downregulation of *Pparg* and *Fabp4* by adipogenic differentiation induction were observed in BMSCs overexpressing *Scara3* (Figure 6B‐E). However, these changes of osteogenic‐ or adipogenic‐related genes expression distinctly diminished when BMSCs were simultaneously transfected with *Scara3* plasmid and *Foxo1* siRNA (Figure 6B‐E). Furthermore, the ARS and oil staining both showed a similar tendency in the four groups (Figure 6F,G). Together, these results indicated that Foxo1 might be a downstream modulator of *Scara3* in the process of adipogenesis and osteogenesis.

### 3.5 Injection of AAV‐*Scara3* into bone marrow cavity alleviated bone loss and MAT accumulation in OVX‐induced osteoporotic mice

**FIGURE 7:**
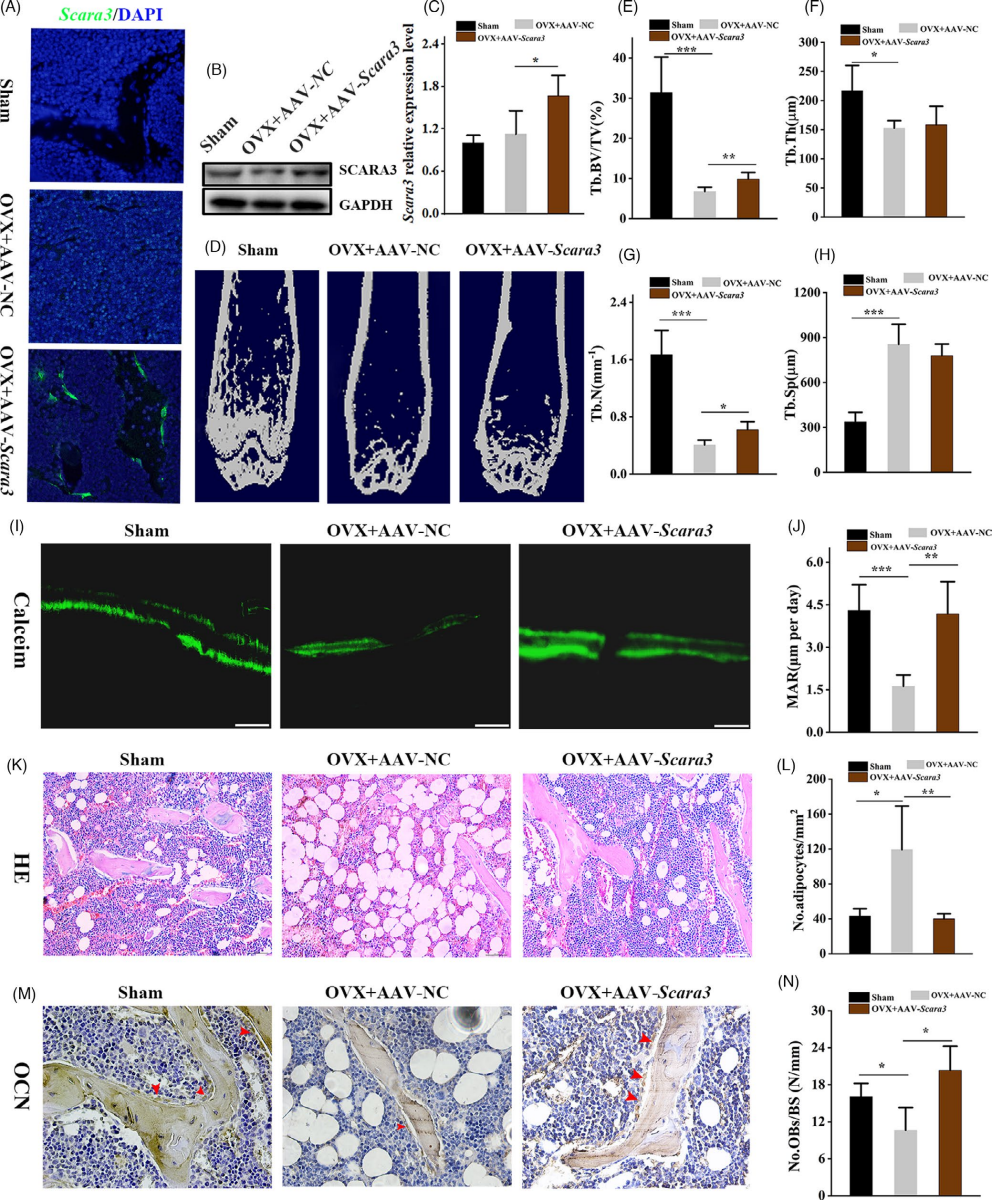
Overexpression of *Scara3* alleviated the bone loss and MAT accumulation in OVX mice. AAVs‐*Scara3* or AAVs‐control was injected into femoral bone marrow cavity of OVX mice. n = 5 per group. (A) *Scara3* distribution in femoral. (B) *Scara3* protein level in femoral BMSCs. (C) Quantification of *Scara3* protein expression. (D) Representative micro‐CT images. (E‐H) Quantitative micro‐CT analysis of trabecular BV/TV (E), Tb. Th (F), Tb.N (G), Tb. Sp (H) bone microarchitecture in femora. n = 5 per group. (I‐J) Representative images and quantitative analysis of calcein double labelling. Scale bar = 100 µm. (K‐L) Representative images of HE staining and qualitative analysis of the number of adipocytes per square millimetre. Scale bar = 125 µm. (M‐N) Representative images of cells with positive OCN staining and the number of osteoblasts per millimetre of bone surface. Red arrowhead indicates osteocalcin‐positive–staining cells. Scale bar = 62.5 µm. n = 5 per group. All data were shown by mean ± SD. *P < .05, **P < .01, ***P < .001, ANOVA

Our results indicated that *Scara3* promoted osteogenesis while inhibited adipogenesis in vitro. Based on that OVX‐induced osteoporotic mice exhibited reduced osteogenic differentiation and increased adipogenic differentiation, we performed OVX surgery in 2‐month C57/B6 female mice to replicate a model of osteoporosis.^35^ Then, these mice were treated with AAVs‐*Scara3* through intra‐bone marrow injection. Immunofluorescence images displayed that *Scara3* was significantly overexpressed in femur, especially to the bone surface (Figure 7A). The BMSCs from mice overexpressing *Scara3* also showed a higher level of *Scara3* expression than the control group (Figure 7B,C). The micro‐CT images exhibited that the bone obtained from AAV‐*Scara3* treated mice has a stronger potential for bone formation when compared to those from control mice (Figure 7D). The quantitative micro‐CT analysis did not show obvious alterations in the trabecular separation (Tb. Sp) and trabecular thickness (Tb. Th) in mice treated with AAV‐*Scara3*. However, a remarkable increase of trabecular bone volume (Tb. BV/TV) and trabecular number (Tb. N) was found in OVX mice injected by AAVs‐*Scara3* (Figure 7E‐H). Moreover, calcein double labelling verified that mineral apposition rate (MAR) was increased in OVX mice treated with AAV‐*scara3* (Figure 7I,J). Overexpression of *Scara3* contributed to the increase of bone formation and decrease of MAT deposition as indicated by HE staining and OCN staining, respectively (Figure 7K‐N). Together, our study showed that *Scara3* restored bone loss partially because of enhancement of bone formation and inhibition of MAT accumulation.

### 3.6 Injection of AAV‐*Scara3* into bone marrow mitigated bone loss in aged mice

**FIGURE 8:**
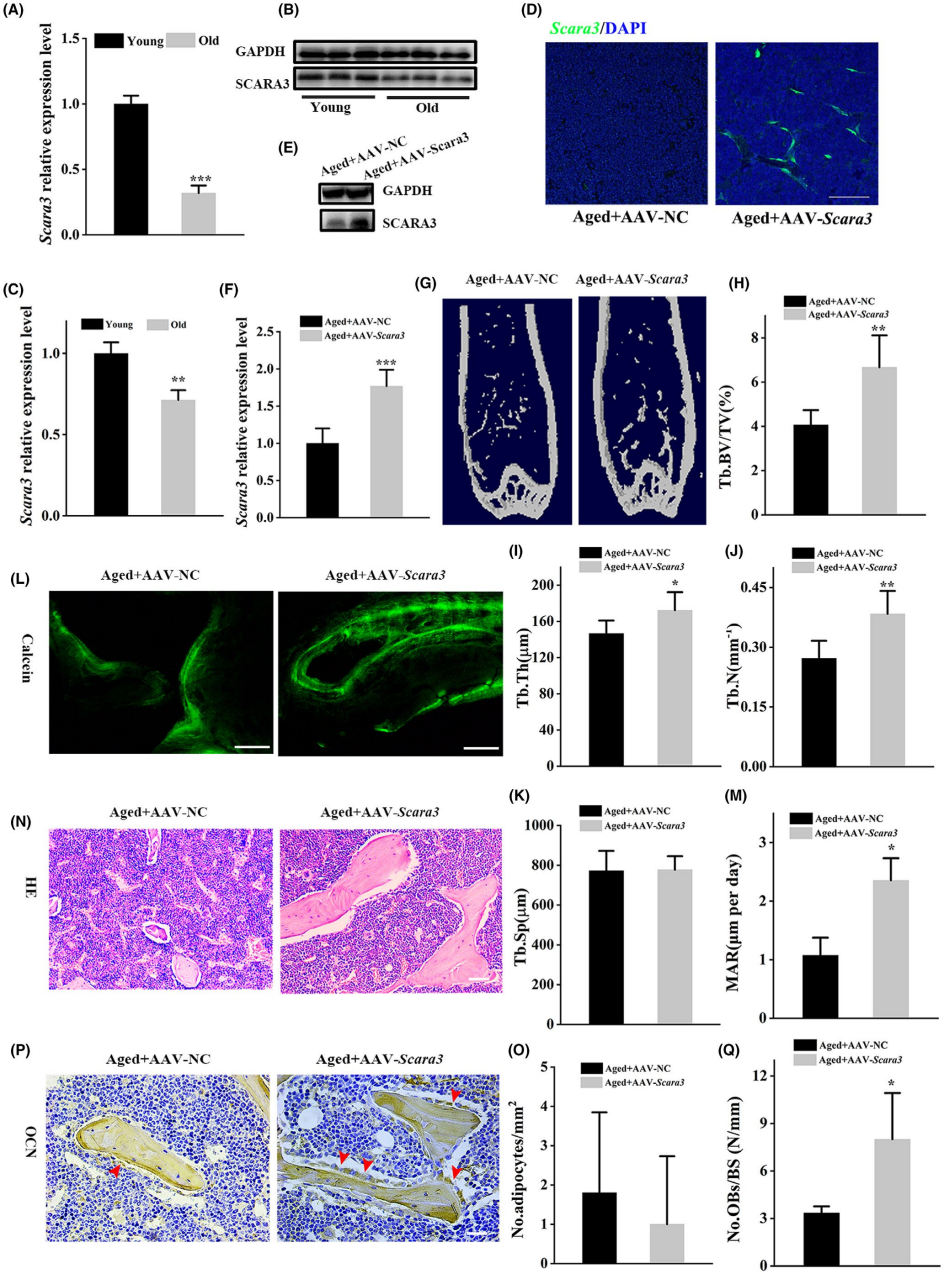
Overexpression of *Scara3* mitigated the bone loss in aged mice. (A) *Scara3* mRNA expression level in BMSCs of young and aged mice. (B‐C) The protein expression and quantitative analysis of *Scara3* in BMSCs of young and aged mice. (D‐S) AAVs‐*Scara3* or AAVs‐control was injected into femoral bone marrow cavity of 15‐mo aged mice. n = 5 per group. (D) *Scara3* distribution in femoral. (E‐F) *Scara3* protein and quantification of BMSCs. (G) Representative micro‐CT images. (H‐K) Quantitative micro‐CT analysis of trabecular BV/TV (H), Tb. Th (I), Tb.N (J), Tb. Sp (K) bone microarchitecture in femora. n = 5 per group. (L‐M) Representative images and quantitative analysis of calcein double labelling. Scale bar = 100 µm. (N‐O) Representative images of HE staining and qualitative analysis of the number of adipocytes per square millimetre. Scale bar = 125 µm. (P‐Q) Representative images of cells with positive OCN staining and the number of osteoblasts per millimetre of bone surface. Red arrowhead indicates osteocalcin‐positive–staining cells. Scale bar = 62.5 µm. n = 5 per group. All data were shown by mean ± SD. *P < .05, **P < .01, ***P < .001, Student's *t* test. [Correction added on 26 July 2021, after first online publication: Figure 8 was a duplicate of Figure 7. It has now been corrected.]

Notably, we revealed a higher expression of *Scara3* in young mice than in old mice at mRNA level and protein level (Figure 8A‐C). In parallel, we also observed the bone phenotypes in aged mice caused by overexpression of *Scara3*. The 15‐month aged mice were obtained through bilaterally intra‐bone injection of AAVs expressing *Scara3*. Injection of AAVs‐*Scara3* into bone marrow caused a significant increase in the level of *Scara3* in femora indicated by immunofluorescence (Figure 8D) and in BMSCs indicated by WB (Figure 8E,F). The micro‐CT images showed that the bone obtained from AAV‐*Scara3* treated mice has more bone mass compared to control mice (Figure 8G). Even though micro‐CT analysis did not identify significant changes in Tb.sp, the Tb. BV/TV, Tb. Th and Tb. N increased 64%, 17% and 41%, respectively (Figure 8H‐K). Calcein double labelling verified that mice overexpressing *Scara3* had a higher MAR than in control mice (Figure 8L,M). Moreover, overexpression of *Scara3* contributed to more numbers of osteoblasts indicated by OCN staining but did not affect the number of adipocytes (Figure 8N‐Q). Taken together, these results suggested that overexpression of *Scara3* through intra‐bone injection of AAVs‐*Scara3* could favour bone formation.

## 4 DISCUSSION

In this study, we revealed that *Scara3* regulated the autophagy signalling and the cell fate switch between osteoblasts and adipocytes. In vitro studies confirmed that *Scara3* enhances osteogenic differentiation at the expense of adipogenic differentiation. Mechanically, *Scara3* regulates Foxo1 and autophagy flux. In vivo, overexpression of *Scara3* alleviated the bone loss and MAT accumulation. Together, our findings provided a new target for the prevention and treatment of bone loss.

SCARA3 is reported to play a role in the antioxidant process.^13^, ^15^, ^36^ our recent study revealed a higher level of SCARA3 expression of Ad‐MSCs in young mice than in aged mice, which was based on the bioinformatics analysis on a public data set.^16^ It suggested that SCARA3 expression might be dependent on the age alteration. Consistent with this result, we found that the expression of *Scara3* in young mice was higher than in old mice. SCARA3, a cellular stress respond gene, can be induced by oxidative stress. It is well established that the level of oxidative stress tended to increase with ageing.^37^ Of note, however, we found SCARA3 expression declined in BMSCs from aged mice. It might be related to the excessive oxidative stress stimulation, chronical stimulation in vivo or the dysfunction of antioxidant induction in aged individuals.

Autophagy level is involved in the bone homeostasis and altered during ageing.^21^, ^38^, ^39^, ^40^ In this study, the deletion of *Scara3* in BMSCs inhibited autophagy‐related signalling while the overexpression of *Scara3* stimulated autophagy‐related signalling. Together, we confirmed that *Scara3* had a positive effect on autophagy in BMSCs. Various autophagy inducers were reported to promote bone formation, while autophagy inhibitors can accelerate bone loss. Autophagy can also maintain the balance between osteoblasts and adipocytes, which has been confirmed in OVX‐induced osteoporosis model mice and aged mice.^20^, ^41^ Several studies showed that the autophagy‐involved bone mass alteration was associated with the altered capacity of cell differentiation in BMSCs.^20^, ^25^ For example, the deficiency of *TSC1*, a positive inducer of autophagy, resulted in bone loss through suppressing osteogenic differentiation, elevating the osteoclast differentiation and adipogenic differentiation.^42^ The autophagy receptor *OPTN* controlled the cell fate switch between adipocytes and osteoblasts in BMSCs.^26^ Here, we validated *Scara3* facilitating autophagy. Further study revealed that the deficiency of *Scara3* exacerbated the osteogenesis‐adipogenesis imbalance in BMSCs. Conversely, the overexpression of *Scara3* favoured osteogenesis at the cost of adipogenesis.

FOXOs play essential roles in the process of osteogenesis in MSCs and the early precursors of osteoblasts.^31^, ^32^, ^33^ Some studies showed that activation of *Foxo1* prevents MSCs from differentiating into fat cells while accelerates osteogenic differentiation of MSCs. Conditional deletion of FoxO1, 3 and 4 in 3‐month‐old mice resulted in osteoblast apoptosis and a decrease of bone formation through the upregulation of oxidative stress in bone.^33^, ^43^, ^44^ Based on that Foxos transcriptional factors, including FOXO1 and FOXO3, acted as crucial inducers of autophagy, we focussed on their expression in BMSCs after the interference of *Scara3* siRNA or *Scara3* plasimid.^34^ Through conducting co‐expression analysis of *SCARA3* with *FOXO1*, followed by the validation of WB and immunofluorescence staining, we suggested that *Scara3* promoted Foxo1 expression. In addition, as expected, we found that *Scara3* promoted osteogenic differentiation and inhibited adipogenic differentiation in BMSCs. However, this effect was impaired by the silence of *Foxo1*. Thus, our study displayed that *Scara3* exerting an effect on the cell fate switch was mediated by Foxo1.

The expression of *SCARA3* gene was previously reported to be associated with bone mass in osteoporotic women bone tissue.^19^ The downregulation of *SCARA3* transcription in osteoporotic bone implied that *SCARA3* might be a key modulator to restore bone loss. Thus, we investigated the role of *Scara3* in bone formation in vivo. The mice overexpressing *Scara3* were applied through intra‐bone marrow injection of AAVs‐ *Scara3*. Next, the *Scara3* expression was tested in BMSCs isolated from femurs of different groups which confirmed that *Scara3* was overexpressed in BMSCs. In consistent with our hypothesis, OVX mice with overexpression of *Scara3* exhibited an increase of adipocytes and a decrease of osteoblasts, which suggested that *Scara3* attenuated bone loss and MAT deposition. Similarly, in the aged mice, the bone loss was alleviated by *Scara3*. Even though various studies found MAT accumulation in aged mice, some studies showed the absence of adipocytes in bone marrow of 12‐month wild‐type mice and over 1‐year TR4^+/+^ mice.^45^, ^46^ The variance of adipocytes numbers in bone marrow might be related to the different lifestyle of diet, exercise or individuals difference. In this study, we did not notice obvious changes of adipocytes by AAVs‐*Scara3* in aged mice, which might be because of the absence of adipocytes in the control aged group. Together, based on the in vitro and in vivo experiments, this study revealed that SCARA3 is a potential target to prevent bone loss and bone adiposity.

## CONFLICT OF INTEREST

The authors declare that they have no competing interests.

## AUTHOR CONTRIBUTIONS

Hui Peng designed the study, and Hui Peng and Peng Chen performed most of biological experiments; Biao Hu, Lin‐Qi Xie and Zhu‐Ying Xia fed the animals and collected the samples; Hui Peng wrote the manuscript; Hui Peng, Zhu‐Ying Xia and Tie‐Jian Jiang carried out the data analysis and revised the manuscript. All authors reviewed and approved the final manuscript.

## Supporting information

Fig S1

Supplementary Material

## Data Availability

The raw data to analyse the genes co‐expression are available in the GTEx database (https://www.gtexportal.org/). The raw data for Scara3 expression in osteoblasts at different stages are available in the BioGPS database (http://biogps.org/#goto=welcome). Other data that support the findings of this study are available within the article or available from the authors upon request.
