# Supplementary material for: Scara3 regulates bone marrow mesenchymal stem cell fate switch between osteoblasts and adipocytes by promoting Foxo1
Source: Cell Prolif. 2021 Jul 12;54(8):e13095. doi: 10.1111/cpr.13095 (PMC8349663; doi:10.1111/cpr.13095)
Supplement: Supplementary file 2 — Supplementary Material [file CPR-54-e13095-s002.docx]

**Figure S1. *Scara3* expression in osteoblasts at different stages.**

The expression of *Scara3* in osteoblasts at different stages including day 5, day 14 and day 21. Each group n=2. All data are expressed as mean ± SD. *P<0.05, ***P<0.001, ANOVA.
